# Evolution of behavioural control from chordates to primates

**DOI:** 10.1098/rstb.2020.0522

**Published:** 2022-02-14

**Authors:** Paul Cisek

**Affiliations:** Department of Neuroscience, University of Montreal CP 6123 Succursale Centre-ville, Montréal, Québec, Canada H3C 3J7

**Keywords:** vertebrate evolution, ethology, sensorimotor control, affordances, action maps, phylogenetic refinement

## Abstract

This article outlines a hypothetical sequence of evolutionary innovations, along the lineage that produced humans, which extended behavioural control from simple feedback loops to sophisticated control of diverse species-typical actions. I begin with basic feedback mechanisms of ancient mobile animals and follow the major niche transitions from aquatic to terrestrial life, the retreat into nocturnality in early mammals, the transition to arboreal life and the return to diurnality. Along the way, I propose a sequence of elaboration and diversification of the behavioural repertoire and associated neuroanatomical substrates. This includes midbrain control of approach versus escape actions, telencephalic control of local versus long-range foraging, detection of affordances by the dorsal pallium, diversified control of nocturnal foraging in the mammalian neocortex and expansion of primate frontal, temporal and parietal cortex to support a wide variety of primate-specific behavioural strategies. The result is a proposed functional architecture consisting of parallel control systems, each dedicated to specifying the affordances for guiding particular species-typical actions, which compete against each other through a hierarchy of selection mechanisms.

This article is part of the theme issue ‘Systems neuroscience through the lens of evolutionary theory’.

## 1. Behavioural control systems

Although behaviour is commonly described as either driven by internal goals or external stimuli, both of these can be considered as two aspects of a single function common to any living thing—the control of its state in the environment. For a living system to remain alive, it must maintain itself within desirable states and counter perturbations away from those states. This is accomplished by physiological control, which operates through feedback loops within the body [1], as well as by behavioural control, which extends through the environment [2–6]. From this perspective, the fundamental function of the brain is not to build knowledge about the world, but rather to complement and counteract the dynamics of the world such that the entire organism-environment system stays within desirable states and away from undesirable ones. When a perturbation away from desirable states is caused by events in the world (e.g. the appearance of a predator), we call that ‘stimulus-response behaviour’. When the perturbation is caused by internal changes (e.g. a growing hunger), we call that ‘goal-directed behaviour’. In both cases, the fundamental organization is a feedback system that controls the animal's state. While these statements are admittedly rather obvious, they are important when discussing complex behaviour because they provide the context without which the adaptive value and the meaning of the whole interaction is obscured [2,4,7].

**Figure 1.**
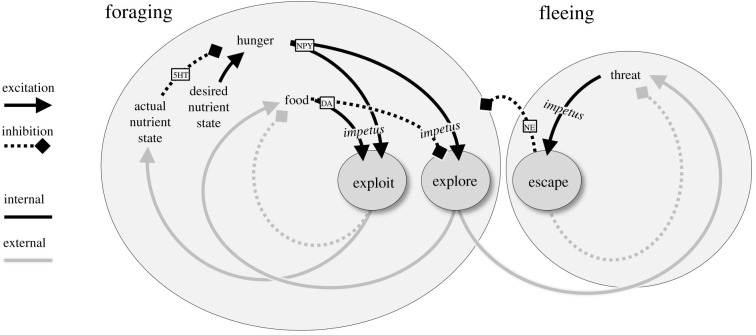
A simple set of behavioural control loops, each involving sensory-to-motor processes internal to the organism (black lines) and motor-to-sensory consequences that take place in the external world (grey lines). The dark circles represent actions motivated by specific conditions (their ‘impetus’). For example, the impetus for exploitation actions is the conjunction of hunger plus the presence of food. Note that in some cases, the consequence of an action is indirect—for example, exploration does not necessarily lead to encountering a threat, but it does increase the chances. Small boxes indicate some relevant chemical signalling pathways (see table 1 for abbreviations).

From the perspective of the feedback organization of behaviour, schematized in figure 1, we can make several functional distinctions. One is the idea that certain conditions motivate actions that result in the reduction or elimination of those same conditions. I refer to these as the ‘impetus’ for action [8]. For example, in the context of feeding behaviour, we can consider two kinds of activity: approaching/ingesting food (‘exploiting’ one's immediate environment) and searching for food elsewhere (‘exploring’ the larger environment). The impetus for ingestion consists of two conditions: (i) the internal nutrient state is lower than the desirable state (i.e. ‘hunger’), and (ii) there is food present. Ingesting the food reduces the ‘hunger + food’ impetus because it improves the internal nutrient state and because it depletes the food. If the food is depleted before the internal state returns to a desirable level (i.e. the animal is still hungry), then the animal enters a new state. Now, the impetus is hunger plus the absence of food, and this motivates different kinds of actions. In particular, it motivates actions that move the animal to other parts of the environment, ideally bringing it into the presence of food. If so, then we return to the previous situation, which again motivates ingestion. Sometimes, of course, one can encounter a threat. This is the impetus for escape behaviour, which again acts as a feedback loop that (hopefully) removes the threat. As shown in figure 1, all of these impetus-action processes take the form of negative feedback loops (the total sign around each complete loop is negative), and this is why they are adaptive. In the language of dynamical systems, the state-to-action pairs (black arrows) complement the action-to-state pairs (grey arrows) in such a way that the desirable state becomes a stable attractor towards which the system tends to flow. Note that in complex systems this attractor is not a point, but an extended manifold within which the state may vary over time. Also note that the interactions between the loops (such as escape inhibiting all foraging-related activity) are of critical importance and make governing behaviour an endlessly expanding challenge. Nevertheless, all behaviour can be seen as involving negative feedback loops with the environment, ensuring that the organism remains in desirable states (e.g. you have a full stomach) and avoids undesirable states (e.g. you are filling someone else's stomach).

**Table 1.** List of abbreviations.

5HT:	serotonin
AC:	auditory cortex
ACC:	anterior cingulate cortex
aIns:	agranular insula
AIP:	anterior intraparietal area
AN:	arcuate nucleus
ANS:	apical nervous system
aOFC:	agranular orbitofrontal cortex
BLA:	basolateral amygdala
BNS:	blastoporal nervous system
BNST:	bed nucleus of the stria terminalis
CA:	Ammon's horn (Cornu Ammonis)
Cbm:	cerebellum
CeA:	central amygdala
CMA:	cingulate motor area
CoA:	cortical amygdala
CP:	caudoputamen
CTh:	collothalamus
DA:	dopamine
DG:	dentate gyrus
dlPFC:	dorsolateral prefrontal cortex
dmPFC:	dorsomedial prefrontal cortex
DPall:	dorsal pallium
Ent:	entorhinal cortex
FEF:	frontal eye fields
FP:	frontal pole
GP:	globus pallidus
IG:	induseum griseum
ILC:	infralimbic cortex
LHA:	lateral hypothalamic area
LIP:	lateral intraparietal area
LPall:	lateral pallium
lPFC:	lateral prefrontal cortex
LS:	lateral septum
LTh:	lemnothalamus
M1:	primary motor cortex
MB:	mammillary body
MCC:	mid cingulate cortex
MIP:	medial intraparietal area
MLR:	mesencephalic locomotor region
MPall:	medial pallium
MS:	medial septum
NE:	norepinephrine
NPY:	neuropeptide Y
OB:	olfactory bulb
PHy:	peduncular hypothalamus
Pd:	pallidum
PHC:	parahippocampal cortex
PMd:	dorsal premotor cortex
PMv:	ventral premotor cortex
PPC:	posterior parietal cortex
PRh:	perirhinal cortex
PT:	pretectum
PTh:	prethalamus
PTub:	posterior tuberculum
PV:	parietal ventral area
Rh:	rhombencephalon
RSC:	retrosplenial cortex
S1:	primary somatosensory cortex
S2:	secondary somatosensory cortex
SC:	caudal somatosensory area
SEF:	supplementary eye field
SMA:	supplementary motor area
SNr:	substantia nigra reticulata
SR:	rostral somatosensory area
Str:	striatum
Sub:	subiculum
T:	tectum
TC:	temporal cortex
TE:	inferotemporal cortex
TEO:	inferotemporal occipital cortex
Th:	thalamus
THy:	terminal hypothalamus
TT:	tenia tecta
V1:	primary visual cortex
V2:	secondary visual cortex
V4:	area V4
V5:	area V5/medial temporal area
VIP:	ventral intraparietal cortex
VLPall:	ventrolateral pallium
VPall:	ventral pallium
VPd:	ventral pallidum
vPFC:	ventral prefrontal cortex
vlPFC:	ventrolateral prefrontal cortex
vmPFC:	ventromedial prefrontal cortex
VStr:	ventral striatum

An important distinction not shown in figure 1 is between two ways in which information from the environment can be used by an animal: (i) for defining the current state (e.g. a threat is present), and (ii) for specifying the metrics of actions that will affect the state (e.g. a direction for escape). Both of these can often come from the same sensory source, but they must be treated differently. For example, information about the presence of a predator should be treated in a categorical fashion, such that regardless of specific details, if something is categorized as a threat then the resulting action should always reduce or eliminate that threat. Ethologists refer to the sensory cues that categorically indicate external conditions motivating specific actions as a ‘key stimulus’ [9] (or ‘sign stimulus’). By contrast, to properly guide escape behaviour one must orient oneself in a direction away from the threat but not in a direction that is obstructed by external objects. This requires information that specifies the potential actions available in the world, or what James Gibson referred to as ‘affordances’ [10].

My goal in what follows is to propose a hypothetical but plausible sequence of the behavioural innovations that occurred in evolution along the lineage that produced humans, framed in the context of feedback control and the concepts defined above, including impetus, key stimulus and affordance. Other concepts often discussed in neuroscience, such as internal representations and goals, will be introduced along the way but always within the context of feedback control, as this makes their functional roles more readily interpretable. Even standard psychological distinctions, such as between perception and action or between cognition and emotion, can best be understood within this general context [4,11,12]. In fact, the overall theme will be that the evolutionary history of the nervous system is essentially a history of the continuous extension of behavioural feedback control further and further into the world [8]. It has often been proposed that viewing the brain from an evolutionary perspective offers a better conceptual foundation than viewing it in terms of human psychological constructs [4,8,13–18], and I will not repeat those arguments here. Instead, I will focus on going ahead and trying to develop some of that evolutionary perspective in order to apply it towards constructing a general hypothesis on the organization of primate brains. In the interest of limiting an already daunting scope, I will focus primarily on food-seeking behaviour and predator–prey interactions, but many of the same mechanisms could in principle apply to other classes of behaviour such as mate seeking.

## 2. A hypothetical phylogeny of affordance-based control

**Figure 2.**
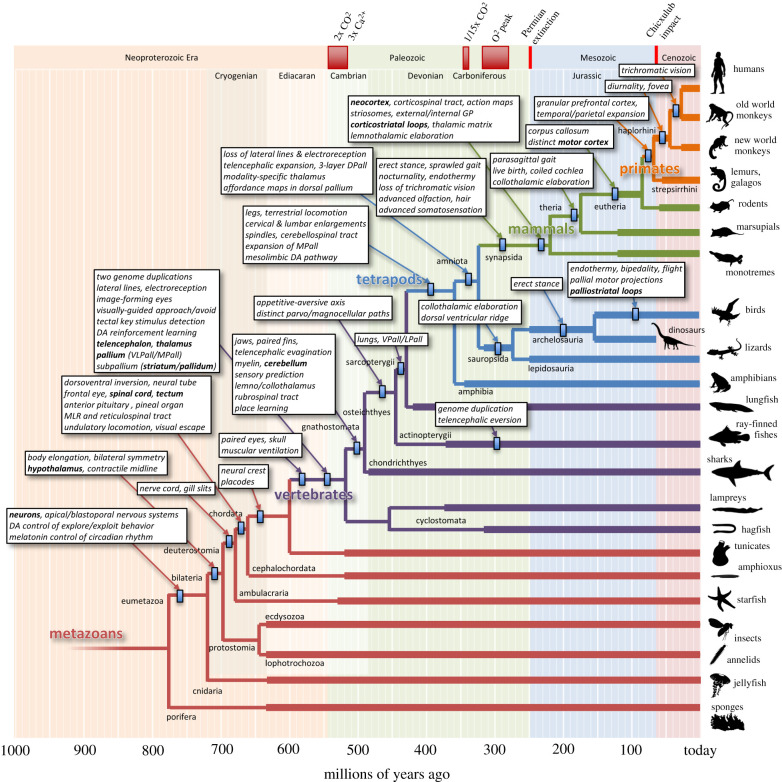
Phylogenetic tree of animals expanded along the lineage that produced humans. Branch points represent some of the key divergences between different lineages, with timing estimates based on the fossil record and molecular clock analyses [19–21]. Thick lines indicate the presence of relevant fossil data (https://paleobiodb.org), and small rectangles indicate the latest estimated timing of the innovations listed in the boxes. Many branch points and lineages are omitted for clarity. The silhouettes of example species are from http://phylopic.org.

Figure 2 shows the phylogenetic tree of animals expanded along the lineage that produced humans, emphasizing some key structural and functional innovations. Note that the ordering of the branches in the vertical direction is arbitrary, and here they are arranged to make room for the boxes describing the innovations of highest interest for the human lineage. This produces what appears on the right edge like a ‘*Scala Naturae*’ from sponges to humans, but it is *not* meant to imply a scale of progress towards higher complexity. In fact, such a tree could be constructed for any species of interest, resulting in a schematic where that species is at the top.

The boxes in figure 2 summarize my current attempt at reconstructing a sequence of evolutionary changes that will be used to guide the rest of this article. In an earlier publication [8], I focused on early stages from the first multicellular animals to stem vertebrates. Here I will only summarize these briefly in order to leave more space to discuss later stages, particularly the transitions from the first terrestrial tetrapods to mammals and then primates.

## 3. Evolution of the vertebrate ‘Bauplan’

Neurons first appeared over 700 million years ago (Ma) as a specialization of epithelial cells of early multicellular animals [22–25]. They formed a diffuse neural net with two specializations: an ‘apical nervous system’ (ANS) rich in chemo- and photo-sensitive cells that communicated through secretions of hormones and peptides, and a ‘blastoporal nervous system’ (BNS) that used synaptic transmission to control contractile behaviour [26,27]. In bilaterians, the body elongated and the ANS and BNS overlapped at one end, where the head and brain would one day appear in some lineages, including chordates, molluscs and arthropods [27,28]. In deuterostomes, the body inverted, placing the neural plate on the dorsal surface [29,30], and then the neural plate folded into a tube, laying down the basic neural plan (Bauplan) of all chordates [31].

**Figure 3.**
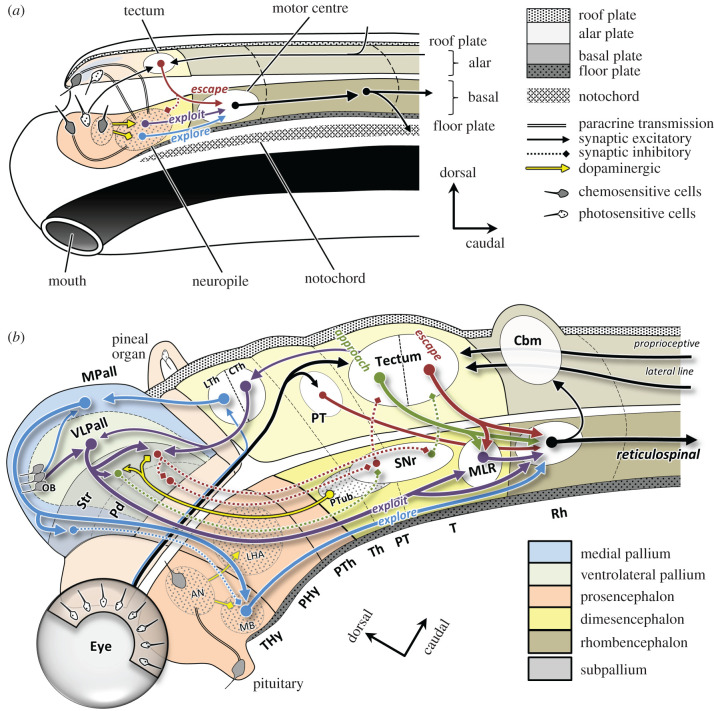
Sagittal views of the basic behavioural circuits of early chordates (*a*) and early jawed vertebrates (gnathostomes) (*b*), based on the prosomeric model [32–34]. Colours indicate proposed homologies. See text for details and table 1 for abbreviations.

Figure 3*a* shows a sagittal view of a hypothetical early chordate based on extensive studies of the lancelet *Amphioxus* [32,35–41]. The neural tube consists of an anterior section that is roughly homologous to the vertebrate hypothalamus and contains a variety of receptors inherited from the merged ANS/BNS, and a posterior section that roughly corresponds to the vertebrate hindbrain and spinal cord [32]. As in nearly all eumetazoans, foraging behaviour was modulated by nutrient intake signalled by the neurotransmitter dopamine [42,43]. High-nutrient intake led to high levels of tonic dopamine and activity patterns that tended to keep the animal ‘exploiting’ rich local regions. Conversely, when nutrient intake fell so did levels of dopamine, leading to long-range ‘exploration’ behaviour. An additional behaviour, ‘escape’, was initiated by tactile or visual input to a dorsal (alar) structure (a putative precursor of the vertebrate tectum [44]), which projected to a ventral (basal) motor centre that initiated fast locomotion away from threats [45], presumably homologous to the mesencephalic locomotor region and reticulospinal tract. These anatomical circuits implement the behavioural control illustrated by black lines in figure 1.

Figure 3*b* proposes how these chordate circuits may have been elaborated in early vertebrates [8]. First, the single frontal photo-sensitive patch of chordates split into two patches that moved to the sides of the head, becoming the lateral eyes [46,47]. These projected *contra*laterally to the tectum, which projected downstream *ipsi*laterally, resulting in escape behaviour oriented away from the threat, like Braitenberg's classic ‘vehicles’ [48]. Further expansion of the eyes and tectum created a spatial map for precise escape behaviour, a key factor in our ancestors' survival during the explosion of predatory activity during the early Cambrian [49,50]. An additional specialization appeared later, whereby a rostral part of the tectum (sensitive to visual space in front of the animal) projected downstream *contra*laterally, thus implementing oriented ‘approach’ behaviour towards objects with specific colour, shape and motion properties, helping our ancestors become effective predators themselves. These circuits have been conserved in all vertebrates studied to date, including lamprey [51,52], jawed fishes [53–58], birds [59], amphibians [60] and mammals [61–65].

It is possible at this point to relate these anatomical circuits to the concepts of key stimuli and affordances, which I do within the context of two very different kinds of problems often labelled as ‘decision-making’. First, there is the decision on whether to approach something or to escape from it. In many vertebrate species, this involves simple thresholding mechanisms, whereby weak prey-like stimuli engage the approach circuit while large salient stimuli such as fast looming engage the higher threshold escape circuit, which inhibits approach [51,53]. This is an example of a ‘between systems’ decision [66], specifically between activating either the approach or the escape circuits of the tectum, and it relies on a categorization of the relevant key stimuli and combining them with internal states (e.g. hunger) to produce an impetus for a specific type of action. In other words, if you're hungry and your rostral tectum detects certain visual features that indicate prey, then this provides the impetus for approach and ingestion. However, if visual input activates high-threshold ‘looming’ detectors, then this provides the impetus for escape and inhibits all other action systems.

Within each of these action systems, there is also a second kind of decision—the ‘within system’ decision on where to move—and it unfolds differently within the approach versus escape systems. In particular, the approach system needs to exhibit ‘winner-take-all’ dynamics to choose a single target among many [8], and this can be accomplished through reciprocal inhibition between spatially selective cells, as documented in the retinotectal system of lamprey [51], zebrafish [55] and birds [67]. This sort of competition produces some of the phenomena usually labelled with the term ‘attention’ [68]. By contrast, escape requires a different kind of processing, in which external objects specify avoidance responses that are averaged together to find a route for unobstructed escape [8]. Studies in zebrafish have supported these predictions at the level of behaviour and at the level of relevant midbrain circuits [56] shared with lamprey [51,52]. For both systems, spatial information is combined with the specific intrinsic dynamics of a neural map (winner-take-all in approach, averaging in escape) to specify an action made possible (afforded) by the world (prey to pursue, unobstructed space through which to flee).

Another major innovation of early vertebrates was the expansion of the alar section of the second segment of the hypothalamus into what would ultimately become the telencephalon [69,70]. It consisted of an outer part called the pallium, which ultimately gives rise to many structures including the cerebral cortex and hippocampus, and an inner part called the subpallium, which becomes the basal ganglia. Much of the history of vertebrate evolution is the history of how these regions expanded, subdivided and specialized in parallel with an increasingly diverse behavioural repertoire. At the early vertebrate stage one can distinguish four subdivisions of the pallium [71,72], which for simplicity, I will provisionally group into two. The first is a ventrolateral segment, ventrolateral pallium (VLPall)—consisting of what is normally called ventral pallium (VPall), lateral pallium (LPall) and dorsal pallium (DPall) [73]—which would later form many parts of the forebrain (in mammals including the neocortex, piriform cortex, claustrum and parts of the amygdala [73–77]). The second is a medial segment, medial pallium (MPall), which would later become the hippocampal complex [73,78,79]. I propose that these two subdivisions each specialized to support one of the two distinct aspects of foraging behaviour: exploiting the local environment versus exploring elsewhere [8].

Exploiting the local environment was governed by the regions of the pallium I have grouped into the VLPall, which integrated olfactory, somatosensory and visual information [71,72] to compute a sensory state estimate that was then used to selectively disinhibit either approach or avoidance behaviours through downstream projections through the subpallium to the tectum via the substantia nigra reticulata. Importantly, the VLPall and its subpallial counterparts (striatum and pallidum) were able to *learn* the associations between the sensory state and the appropriate action using reinforcement signals in the form of phasic bursts of dopamine from a basal structure called the posterior tuberculum (homologous to the ventral tegmental area and substantia nigra compacta [80,81]). That is, the ancient role of dopamine in signalling the tonic average intake rate was extended to signal phasic increases or decreases of intake that could be used to reinforce adaptive state-action policies and to invigorate appropriate actions when needed [82].

Exploration behaviour, in contrast, was governed by the MPall, which becomes the hippocampal complex [73,78,79]. Like the VLPall, it also integrated olfactory and visual information and also projected through a subpallial circuit (homologous to the lateral and medial septal nuclei) and the basal hypothalamus (ventromedial shell and mammillary bodies) [83]. However, because its downstream projections induced long-range locomotion, it did not learn the cues for approaching a particular object but instead learned gradients, such as odour plumes, that would bring the animal to a richer locale [78,84–87].

Shortly after the divergence of jawless fishes (agnathans) and jawed fishes (gnathostomes) over 500 Ma, two other innovations appeared along our lineage. One involved the parcellation of the thalamus into a portion called the ‘lemnothalamus’, which received visual information directly from the retina and projected primarily to the MPall, and a portion called the ‘collothalamus’, which received visual information from the tectum and projected primarily to the striatum but also to the VLPall [72,88,89]. The former pathway presumably aided navigation by providing information for learning visual landmarks [87], while the latter, I propose, carried information that could be used to learn new key stimuli using combinations of the kinds of hard-wired feature detectors already present in the rostral tectum.

The second innovation was still more significant and involved the elaboration of the dorsal part of the anterior hindbrain into what became the cerebellum. According to Montgomery, Bodznick, Bell and others [90–93], this began with inhibitory neurons that cancelled out signals shared across multiple pressure receptors (akin to ‘common mode rejection’ in electronic signal processing), resulting in ascending sensory signals that were more sensitive to external events than to the consequences of the animal's own active motion. This was assisted by subtracting out an efference copy of the motor command, effectively implementing the ‘reafference principle’ of VonHolst & Mittlestaedt [94]. Elaboration of this structure with a synaptic matrix turned it into an adaptive filter that could learn to *predict* complex sensory consequences of motor commands, ultimately allowing our ancestors to overcome the challenges associated with moving a large body at fast speeds and to become very effective predators.

## 4. The transition to land

**Figure 4.**
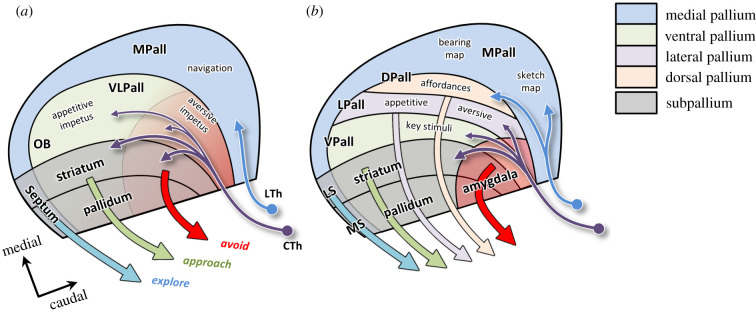
Parcellation of the forebrain in anamniotes (*a*) and amniotes (*b*). In both, the forebrain is shown as a flattened sheet divided into pallial (MPall, VLPall, etc.) and subpallial sectors (striatum and pallidum), receiving lemnothalamic (blue) and collothalamic input (purple). See table 1 for abbreviations.

Figure 4*a* illustrates the forebrain architecture in anamniotes, in which a rostrocaudal gradient can be distinguished in the VLPall and its descending projections. The rostral end of the VLPall and its descending projections through the ventral striatum and pallidum learned to detect key stimuli that motivate appetitive actions such as approach and ingestion. At the caudal end, including the dorsal striatum and pallidum as well as what in mammals forms the amygdalar nuclei and the bed nucleus of the stria terminalis, the VLPall learned key stimuli for avoidance behaviours. Importantly, the actual moment-to-moment control of these behaviours remained largely the province of downstream systems such as the nigrotectal circuits and the locomotor projections to the reticulospinal tract [95]. The major role of the telencephalon was to selectively invigorate those downstream systems on the basis of the animal's estimated state in the world, indicated by the current set of relevant external key stimuli as well as internal ‘interoceptive’ signals. For example, when the animal oriented towards an edible item, the VLPall and subpallium recognized the impetus of ‘hunger + edible item ahead’ and invigorated the approach circuits of the rostral tectum. Conversely, when sensory information indicated danger, it instead invigorated the avoidance circuits. This basic architecture appears to have been retained up to and beyond the transition to land around the end of the Devonian period and can still be found in modern amphibians [96].

The transition to terrestrial life presented our ancestors with a radically novel environment full of new demands and opportunities. Among these were a new impetus, ‘thirst’, and a much more challenging task of regulating body temperature. There were also major changes in sensory information, including the loss of effectiveness of the lateral lines, less reliable directionality of airborne odours, and most importantly, a dramatic increase in the visual range [97,98]. This latter feature of terrestrial life favoured the elaboration of visual landmark processing in the MPall, permitting better navigation. According to the parallel map theory of Jacobs & Schenk [87], navigation involves a ‘bearing map’ that relies on directional gradients and a ‘sketch map’ that relies on information about relative positions of cues that can define landmarks. On land, the sketch map became much more effective because animals could now see very distant landmarks, like trees and mountains, which provide a very stable reference frame for guiding exploration ever further into the world. Thus, animals with a larger MPall and more extensive visual input could seek food over a larger range and were favoured by natural selection. Indeed, modern amphibians are very good navigators [99], and recent studies show that when toads navigate to water using learned allocentric geometric relationships in their environment, as opposed to specific key stimuli, there is an increase of neural activation in their MPall [100].

Figure 4*b* shows further subdivisions of the forebrain. Precursors of these can be identified in a broad class of animals, including lungfish [101], amphibians [96] and even lamprey [71,72], but they become especially distinct in the enlarged brain of amniotes [73]. In particular, at the border between the former VLPall and MPall domains, there appears an island of specialization defined by two signalling gradients: a dorsal-to-ventral signal from the ‘cortical hem’ and a rostral-to-caudal signal from the ‘anti-hem’ [102]. The differential overlap between these signals induces two distinct domains, the LPall and the DPall.

What could have been the functional roles of these subdivisions? Here, I propose that they expanded the ancient functional role of the ancestral VLPall (learning the conditions for activating different tectospinal systems), by specializing towards different types of sensorimotor control. In particular, below I will suggest that the LPall specialized in integrating interoceptive information with external key stimuli to define the impetus for appetitive and aversive actions, while the DPall specialized in detecting affordances. Importantly, however, this specialization of function appears to have proceeded quite differently along the two main branches of amniotes: the sauropsids (reptiles and birds) and the synapsids (mammals and their ancestors).

The sauropsids retained the diurnal lifestyle of early amniotes and continued to rely on increasingly elaborate tectal circuits for visuomotor control. This was accompanied by the expansion of forebrain regions receiving collothalamic projections (striatum, VPall and LPall). The VPall continued to govern learning key stimuli for modulating downstream systems, but it started to become increasingly modality specific, along with the corresponding regions of the thalamus. It also expanded significantly, bulging into the ventricular space to form the prominent ‘dorsal ventricular ridge’, which contains distinct regions specialized for different modalities [103–107]. I propose that these regions were primarily sensitive to the kinds of key stimulus combinations that reliably indicated the presence of foods, mates or threats, thus learning to identify the impetus for releasing specific kinds of tectally guided behaviours via descending striatal projections. In other words, the VPall was still playing the same modulatory role it played in early vertebrates, but it was becoming much more sophisticated.

In contrast with the segregation of sensory modalities in the VPall, the LPall remained largely multisensory. It is difficult to speculate about its role in early amniotes, but some hints may come from studies of the homologous structures in extant descendants: the ‘pallial thickening’ in lizards and turtles, the ‘mesopallium’ in birds, and in mammals, the agranular regions of orbitofrontal, insular and perirhinal cortex as well as the claustrum and the endopiriform nucleus [73,108]. These have been implicated in a variety of integrative functions pertaining to appetitive and aversive behaviour, physiological arousal and responses to startling stimuli [109–118]. For example, cells in the agranular insula respond to visual cues associated with food rewards, but only if the animal is hungry or if a hunger state is induced through hypothalamic stimulation [114] and cells in the posterior part of agranular insular cortex modulate behavioural and autonomic responses to aversive states [110]. One common theme suggested by many studies is that structures derived from the LPall are involved in combining external and internal information to specify the animal's state over periods of time that are longer than the presence of specific stimuli or environmental configurations. For example, if a predator appears then the animal should escape, using its highly efficient tectal circuits, but then it should remain in a state of heightened sensitivity to similar cues should the predator return. One is tempted to call this ‘fear’ or ‘anxiety’. If we are willing to extrapolate from studies of mammals, we can suppose that the amniote LPall retained the appetitive-aversive axis of early vertebrates, with the anterior end specialized for estimating the impetus for appetitive behaviours and the posterior end specialized for learning to identify cues that motivate escape, freezing and other defensive actions [109,110,113,114,119]. However, it should be noted that this distinction is blurred by the extensive interconnections among pallial regions, particularly in what in mammals forms the basolateral amygdala and in birds the caudal nidopallium [120].

What about the DPall? In contrast with the visual region of the VPall, which receives information via the optic tectum and collothalamus, the visual area in the reptilian DPall (called dorsal cortex) receives retinal information directly through the lemnothalamus [89,121]. This is similar to the adjacent MPall, which relies on information about the relative position of landmarks for use in navigation. Relational processing of a different sort also occurs in the DPall, but not for landmark detection. Instead, I propose that visual information in the DPall was used to detect the *affordances* of the immediate environment (what is climbable, what is a good place to rest, what is a good place to hide, etc.), and downstream projections from this region helped to select from among the component sensorimotor interactions necessary for moving around the world. In other words, I am proposing that the lemnothalamic information used for navigation in the MPall extended into the DPall to create a telencephalic region that assisted some of the tectal guidance systems for a more diverse class of behaviours (see Aboitiz *et al*. [122] for a similar proposal).

What is the evidence for this? At first glance, the data seem to contradict my conjecture. In particular, thalamic projections to the dorsal cortex of reptiles have very limited spatial topography [123,124], certainly not enough to form even rudimentary images. That seems like a death-knell for any theory of affordance detection. How can you guide movement around in space if you cannot even build a map of visual space? The answer I would propose is that one does not need a map of visual space in the *pallium* in order to guide movement through space, because one already has an excellent map in the *tectum*. That is, the actual visual guidance of locomotion (precisely aiming in a specific direction, avoiding obstacles) continued to be controlled by the retinotectospinal circuits as it had been for millions of years. What the DPall added to this was to help select when and whether one should do so, by releasing the tectal locomotor circuits at the right time and in the right context via modulatory pathways through the subpallium. To fulfil that role, it did not need to build an image of visual space; it just needed to be sensitive to the combinations of features that reliably indicate the presence of a particular locomotor affordance and link its detection with the appropriate behavioural context.

**Figure 5.**
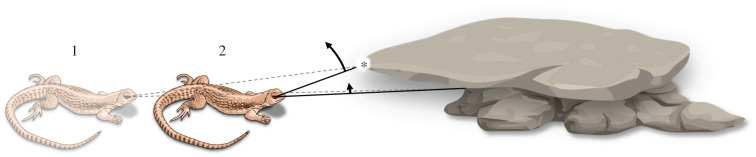
Scheme for detecting ‘hideability’. As the animal approaches a potential hiding spot by moving from point 1 to point 2, the projections of the environment on its retina shift (from dotted to solid lines). This produces a particular pattern of optic flow across the visual field: the texture above a certain point (*) is brighter and moves away from the centre of the field more quickly (long arrow) than the texture below, which is darker and expands more slowly (short arrow).

Let us consider ‘hideability’—the potential to provide shelter. As shown in figure 5, as an animal approaches a potential place to hide, there are particular patterns of optic flow that reliably indicate whether that place will indeed afford shelter for that animal. Most significant is a horizontally oriented motion contrast edge, above which texture is brighter and rises upwards quickly and below which texture is darker and moves more slowly. To indicate hideability, that motion edge must be above the point of visual expansion, and ideally it should be just barely above it. Detecting such a combination of features does not require a map of space, or even a visual image. What it requires is rough spatial selectivity combined with motion and brightness selectivity, with the right dependence on geometric transformations (properties quite similar to those for navigating using landmarks); and, of course, all of this should be modulated by ‘alarm’ signals indicating the need to find shelter.

This kind of rudimentary selectivity is indeed found in reptile dorsal cortex. Studies in turtles show that despite the fact that thalamic projections to the visual part of the DPall are not retinotopic, spatio-temporal aspects of visual stimuli can nonetheless be decoded from neural activity recorded therein [125,126]. The reason is that while the receptive fields of individual neurons are large, they are not entirely uniform across space. Furthermore, they are sensitive to brightness edges and their polarity, to visual motion, and respond with different latencies to central versus lateral stimuli [126,127]. Thus, although the reptile DPall may not have the acuity to form images of the visual world, it does possess the right combination of properties necessary to detect the relevant affordances of that world.

Admittedly, my particular example of finding shelter may not be such a good one in the context of data from an animal that carries its shelter on its back, but the idea generalizes to many other animals and many other kinds of affordances, such as ‘climbability’, ‘supportability’ etc., and it makes testable predictions. One is that cell groups possessing the right combination of properties for detecting hideability should be modulated by cues related to threats, like loud noises or fast looming stimuli. Consistent with this, the reptile DPall receives monosynaptic projections from the locus coeruleus [128], a structure often associated with producing general arousal through its diffuse noradrenergic projections [129]. However, to my knowledge, the neurophysiological experiments necessary to test these kinds of predictions have not yet been performed, so the conjecture remains speculative.

In summary, I suggest that early diurnal amniotes expanded their VPall and related pallial amygdala and improved their ability to use olfactory, visual, auditory and somatosensory information for detecting the key stimuli that indicate the conditions motivating a variety of appetitive and aversive behaviours, which themselves continued to be controlled through ancient tectospinal circuits. The LPall integrated such cues with internal signals such as hunger or fear, while the DPall detected the relevant affordances in the world (e.g. a place to hide when threatened). This basic organization was retained in sauropsids as they dominated the diurnal world and elaborated much further in some branches like archosaurs and later, birds. However, given my focus on the lineage that produced humans, I will not discuss sauropsid-specific innovations in any more detail and instead refer readers to recent excellent reviews [73,130,131].

## 5. The retreat into nocturnality

While the sauropsids came to dominate the diurnal world during the Mesozoic, our synapsid ancestors reduced their body size [132], developed the ability to regulate body temperature [133,134] and became nocturnal [135]. This led to a dramatic re-weighing of the role of different sensory modalities in guiding their behaviour. Notably, vision was reduced at both a peripheral and central level. Early mammals lost sensitivity to medium wavelength light [47], becoming effectively red-green colour-blind [136], and they reduced the size of their optic tectum (in mammals called the superior colliculus) along with its collothalamic targets in the VPall, LPall and striatum. Instead of relying on vision, they elaborated the olfactory, auditory and somatosensory systems [95]. In fact, most of the pallium of early mammals consisted of the VPall, or the piriform cortex [137], which became dominated by inputs from a highly elaborate olfactory system. Early mammals also significantly improved their somatosensory abilities. The hair covering their bodies, in addition to insulation, provided an expanded tactile sense, particularly thanks to the elongated vibrissae near the snout [138]. In the dark, this became a major source of information on our ancestors' immediate surroundings, providing clues about the layout of obstacles, surfaces and apertures through which one could pass. In short, it became a major source of information for detecting affordances, favouring further expansion of the DPall [102].

In mammals, the DPall becomes the neocortex, and its organization is quite unusual [95]. It develops in an inside-out fashion into a six-layered structure that receives thalamic input radially, forming columns. This is quite unlike the homologous dorsal cortex of reptiles, which has only three layers, develops from the outside-in (with a few exceptions) and receives thalamic inputs that travel through it tangentially. The radial thalamic projections to mammalian neocortex make it possible for it to expand without incurring excessive connection costs, and so it has—in many mammals ballooning out to cover up almost all of the rest of the brain.

Alongside the expansion of the neocortex, early mammals introduced two other major innovations. First, they elaborated projections from the neocortex to downstream and spinal centres, allowing more direct and fine-tuned control of interaction [139,140] (although there is an ongoing debate as to the extent to which such descending control was novel to mammals and birds [95,141] versus an elaboration or duplication of ancestral pathways found in non-avian reptiles [105,142,143], amphibians [144], sharks [145] and lamprey [146]). Second, mammals introduced an additional output of the pallidum that, instead of projecting downstream, projected to a ventrolateral subdivision of the thalamus, which itself projected back to the neocortex [89,95]. This pathway created a recurrent circuit from each cortical subregion to a specific striatal target, to a specific pallidal site, to a thalamic target and back to that same cortical subregion [147,148]. In this way, the ancestral subpallial mechanisms for selectively invigorating midbrain sensorimotor circuits now could selectively invigorate *cortical* sensorimotor circuits [149]. With these two innovations, early mammals effectively shifted much of the control of behaviour from the visuomotor midbrain to the somatomotor forebrain, setting the stage for a diversification of their behavioural repertoire.

**Figure 6.**
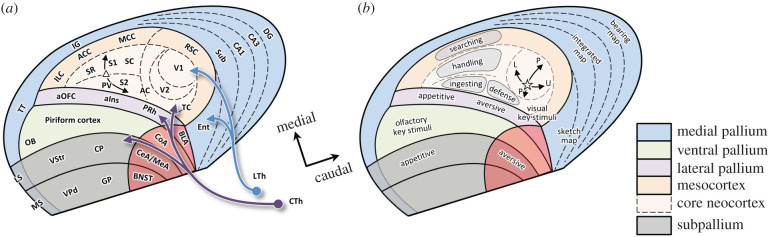
Topological schematic of forebrain organization in early mammals. (*a*) Anatomical subdivisions in a hypothetical early mammal, in which the DPall/neocortex consists of a central core (light tan) that includes somatomotor, auditory, and visual regions, surrounded by a ring of agranular mesocortex (dark tan) and lateral pallial regions (violet). The somatomotor area includes maps of the body in which the mouth/nose region lies at the point marked with a triangle and trunk and limb regions extend both medially and caudally along the arrows. (*b*) Proposed functional subdivisions for different kinds of actions (shaded regions). The star indicates central visual space with arrows indicating lower (L), upper (U) and contralateral peripheral (P) visual space. See text for details and table 1 for abbreviations.

In all mammals, cerebral cortical organization can be understood as a set of concentric rings around a central core of six-layer ‘eulaminate’ cortex [74,83,150–154], as shown in figure 6. That core is surrounded on the medial side with agranular ‘mesocortex’ defined by a gradient of morphogens emanating from the cortical hem, and on the lateral side with regions derived from the LPall, defined by a gradient emanating from the anti-hem [151,153,154]. This ‘dual’ nature of neocortical organization has been discussed for decades [150], and although it has sometimes been proposed as a theory of sequential outer-to-inner evolution [155], comparative data suggest that all of these regions existed in early mammals and diversified in parallel [17,137]. Nevertheless, the developmental gradients are paralleled by gradients of connectivity [152,153], making it possible to divide the neocortex into two sheets [121,156,157]: (i) a dorsomedial region that receives lemnothalamic input like the adjacent MPall, is spatially topographic and is strongly interconnected with the dorsal striatum; and (ii) a ventrolateral region that receives collothalamic input like the adjacent LPall, is non-topographic and is strongly integrated with the ventral striatum, ventral and lateral pallia, and the hypothalamus.

Figure 6*a* shows a simplified schematic of the forebrain organization in the putative last common ancestor of mammals. The parcellation of neocortical regions is based on studies in marsupials [158–161] and their comparison with monotremes [162,163] and placentals [164–166]. In particular, note the central ‘core’ island of neocortex (light tan), bordered on the medial side by agranular mesocortical regions (dark tan) corresponding to infra/pre-limbic, anterior cingulate, mid-cingulate and retrosplenial cortex, and on the lateral side by regions derived from the LPall (violet) including the agranular orbital, agranular insular and perirhinal cortex [74,151]. That central island was dominated by somatosensory input rostrally, visual input caudally, with auditory input arriving at an intermediate site.

The somatosensory region included five maps of the body, three located medially (rostral somatosensory area (SR), primary somatosensory cortex (S1), caudal somatosensory cortex (SC), which roughly correspond to areas 3a, 3b, and 1–2 [162,167]) and two laterally (parietal ventral area (PV), secondary somatosensory cortex (S2)). These maps converged at a site sensitive to the mouth and nose (marked by a triangle) and represented more distal and caudal parts of the body medially as well as caudally, as indicated by the arrows. Note the absence of a distinct motor cortex. Indeed, comparative evidence suggests that early mammals did not possess a separate primary motor cortical region but rather a somatomotor ‘amalgam’ [168], which differentiated into primary motor and somatosensory strips in placental mammals. Furthermore, it is important to recognize that even in modern mammals all of these regions still retain both sensory and motor roles [164]. In particular, while movements can be most easily evoked via microstimulation in primary motor cortex (M1), they can also be evoked from somatosensory and parietal regions [164,169]. Of these, area 3a has the lowest thresholds for evoking movement, suggesting that M1 may be an elaboration of its rostral part, within what in figure 6*a* is labelled as area SR.

The visual region included a primary visual cortical area receiving lemnothalamic input as well as secondary and temporal visual areas receiving collothalamic input [170,171]. This collothalamic input to the DPall is particular to mammals, implying a shift of input from neighbouring VPall and LPall [121], and presumably contributing to the ventrolateral non-topographic sheet described above [122,156,157].

What could be the functional interpretations of this ancient organization? Figure 6*b* presents a proposal based on the notion that the ancestral role of the DPall was the detection of affordances, as suggested above for early amniotes, which expanded as mammals adapted to a nocturnal lifestyle. For example, consider the challenges facing a small furry animal searching for food in the darkness. The impetus motivating general foraging actions would involve odour gradients (coupled with hunger), sensed by a VPall that became almost exclusively olfactory. By contrast, the specification of affordances for moving around in space to approach and obtain the food came from somatosensory information provided by the rich tactile input processed in the DPall/neocortex. This information could be used for guiding several distinct types of foraging-related behaviours, roughly classifiable into searching, handling and ingesting.

Searching involves a variety of locomotor patterns involving the trunk, forelimbs and hindlimbs. It is motivated by odours but specified by tactile as well as some visual information, just like the locomotor affordances of the ancestral DPall in amniotes. I propose that in early mammals, it was governed by the medial agranular regions including the somatosensory mid-cingulate cortex and the visual retrosplenial areas. This predicts the existence of a cingulate somatomotor region in ancestral mammals, corresponding to the cingulate motor area found in primates. Ingesting, on the other hand, is quite different. It strongly involves the mouth and nose, sometimes the forelimbs, and is motivated by local cues from the vibrissae, nose and tongue, as well as olfactory and visual information on potential food items, modulated by hunger. I suggest that it was governed by lateral regions of the neocortex, including the dysgranular PV map and the anterior (appetitive) parts of the LPall, which would become the agranular parts of orbitofrontal and insular cortex. A similar proposal was made previously by Aboitiz & Montiel [154]. In addition, between the task of searching and the task of ingesting, there is another task—‘handling’, which involves various species-typical behaviours that make food ingestible (such as burrowing and digging out a root or a nest of insects, or biting down on a nut to crack its outer shell). Like ingesting, it heavily relies on the mouth and nose but also on other parts of the body and involves specifying potential actions using a mixture of tactile and visual information. I suggest that this was governed by the cortical regions lying between the cingulate and dysgranular insular areas, corresponding to area S1, which includes a map of the entire body, as well as the adjacent SR and SC [159]. Finally, a region governing another class of behaviour, including defensive actions such as freezing, was located caudo-laterally near the auditory cortex, area S2 and perirhinal LPall. In summary, the expanding neocortex of early mammals contained a set of ‘action maps’ specialized for different species-typical behaviours [172].

With the growing number of these different behavioural patterns, the challenge of arbitrating among them grew. This is similar to the ‘between systems’ decision problem discussed above in the context of subpallial signals that selectively invigorate different tectal circuits for approach and avoidance. In mammals, with their subpallial projections through the thalamus back to the neocortex, the same architecture could be used to selectively invigorate different *cortical* action maps, i.e. selecting the type of activity in which the animal will engage at a given time [66,149]. In agreement with this, single-neuron reconstructions show that thalamic regions receiving basal ganglia input innervate long rostrocaudal strips of cortex [173], potentially activating an entire behavioural action map (shaded regions in figure 6*b*), and microstimulation [174] and anatomical tracing studies [175] suggest that the separate action maps compete against each other through long-range inhibitory connections. This architecture could allow the basal ganglia to arbitrate between different types of behaviours [149], and once one is selected, the ‘within system’ choice of a specific movement could be resolved in the appropriate cortical regions themselves [165,176].

Consider again our nocturnal insectivorous ancestor. If hungry, it uses the circuits of its MPall/hippocampal complex to navigate to an area where food can be found. Once there, it invigorates cingulate circuits governing more local search actions specified by a map of walkable space in retrosplenial cortex interconnected with cingulate motor regions, ultimately bringing it to a promising site (e.g. a fallen tree trunk). Here, the prediction of potential rewards learned through secondary reinforcement motivates dorsal circuits for handling behaviours such as burrowing with the forelimbs while sniffing the exposed material. If this leads to uncovering a nest of insects, prediction of direct primary reinforcement now shifts behaviour towards feeding and ingesting, using the lateral ingestion system guided by learned appetitive cues. If the food is depleted before hunger is satisfied, more handling is engaged, and if this still fails to yield primary rewards, the animal shifts back to searching or to navigating elsewhere. Selection between all of these types of activities is made via selective invigoration through the basal ganglia, biased by signals from frontal regions of the mesocortical ring. In particular, the topologically lateral regions, including agranular orbitofrontal and insular cortex, can bias behaviour towards the object to which the animal is oriented, ‘accepting’ what it offers, while the medial regions, notably including the anterior cingulate cortex, can bias behaviour towards ‘rejecting’ that offer and searching elsewhere [177–179].

In addition to selecting between different types of activities, decisions must also be made within the scope of the chosen activity. These are ‘within system’ decisions between different objects to approach, different places to dig, or different items to eat. They are not made at the level of the basal ganglia loops, but within each specific and idiosyncratically organized cortical map of space: distant space for searching and orienting, space near the front of the body for handling, and space close to the head/mouth for ingesting [13]. Cells within these maps are tuned to a particular set of parameters defining different actions, and they compete against each other such that groups of cells defining similar actions co-excite while groups defining dissimilar actions mutually inhibit each other [180]. The result is a winner-take-all process similar to the approach system of the rostral tectum, but now unfolding within a specific cortical map of the affordances pertinent to a given class of species-typical action.

To summarize, behavioural control in early mammals consisted of parallel sensorimotor loops that were selected at two hierarchical scales [165]: (i) a subpallial/basal ganglia mechanism for selectively invigorating a specific cortical action map [149,181], and (ii) for the selected map, the cortical dynamics that specify and select one action among others [66,176]. The neocortex expanded during evolution as mammals diversified their behavioural repertoire, making it possible for them to invade a wide variety of niches, including returning to the seas, migrating into extreme climates, and finding food and safety in the branches of trees.

## 6. The innovations of primates

**Figure 7.**
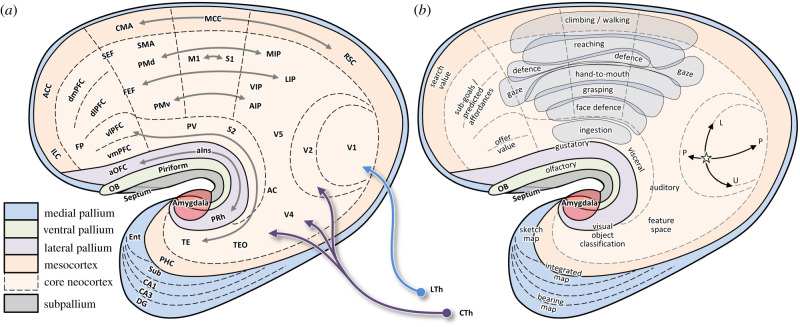
Topological schematic of forebrain organization in anthropoid primates. (*a*) Anatomical subdivisions (same format as figure 6), with the neocortex expanding and curving around the insula and subdividing into many new distinct areas. Grey arrows illustrate the reciprocal pattern of fronto-parietal and fronto-temporal connections. (*b*) Proposed functional specializations, particularly emphasizing the expansion of fronto-parietal cortex to support a diverse range of species-typical actions (shaded regions). See text for details and table 1 for abbreviations.

Figure 7*a* illustrates how the basic mammalian organization of figure 6*a* became elaborated in primates, which evolved within an arboreal niche in the small branches of angiosperm trees [182] and later diversified to include descendants that returned to a diurnal lifestyle. Their success in arboreal life was enabled by a number of key adaptations, including a grasping and leaping form of locomotion, frontally facing eyes, and a brain that in extant species is significantly larger than that of other similar size mammals [16,183]. This involved expansions of frontal, parietal and temporal regions, causing the entire cerebral cortex to curve around the insula into the familiar C-shape of primate brains, bringing the hippocampus and amygdala around to the medial wall of the temporal lobe.

Note the significant expansion of the somatomotor region (particularly of what I associated above with ‘handling’ behaviours) into a wide strip now subdivided into an inner circuit of reciprocally connected M1 and S1 as well as an outer circuit of reciprocally connected premotor and parietal areas [184]. This expansion pushed areas PV and S2 up against the dysgranular insular cortex where gustatory and visceral regions are found and greatly expanded the parietal territory between primary somatosensory and visual areas [185]. What was the functional significance of this expansion?

Figure 7*b* illustrates the proposal, first described by Michael Graziano and colleagues [172,186], that the fronto-parietal circuit implements a set of action maps for guiding species-typical actions such as walking, reaching, grasping, bringing objects to the mouth, orienting the head and eyes, and defending oneself against threats. While originally controversial, this proposal has now been supported by microstimulation and inactivation studies replicated in several laboratories and across a wide variety of species, including macaques [172,186–188], galagos and squirrel monkeys [165,175,189,190], tree shrews [167] and rodents [191]. In particular, the similarity of the maps observed in macaques and galagos [165] suggests that this organization emerged at the root of primates about 70 Ma [192]. In the context of the hypothetical brain organization of ancestral mammals shown in figure 6*b*, I propose that the expansion of primate fronto-parietal cortex primarily involved the circuits formerly responsible for ‘*handling*’ activities, making possible the many highly diverse ways of interacting with the environment of which primates are capable.

Each of these types of interactive activities engages a dedicated fronto-parietal system that controls a specific set of bodily effectors (hindlimbs for leaping, forelimbs and hindlimbs for climbing and walking, forelimbs and head for manipulation and bringing objects to the mouth, etc.), and each requires sensory information to be transformed into a specific and idiosyncratic reference frame. For example, reaching actions involve the medial intraparietal cortex (MIP) [193,194], which represents targets within reach with respect to the direction of gaze and the position of the hand [195,196] and which is interconnected with frontal regions controlling reaching, such as dorsal premotor cortex (PMd) [194,197–199]. Grasp control involves the anterior intraparietal area [200], which is sensitive to object shape and is interconnected with grasp-related frontal regions such as the ventral premotor cortex [201]. The control of gaze involves the lateral intraparietal area [202], which represents space in a retinotopic frame [203,204] and is interconnected with regions controlling gaze, such as the frontal eye fields (FEF) [205] and the superior colliculus [206]. Each of these systems is sensitive to the particular types of affordances present in the world (objects that can be reached, grasp points around an object, branches that one can walk upon, salient targets to look at, etc.) and specifies the potential actions available to the animal at any given moment.

Because one cannot often perform multiple actions at the same time, selection must take place, and it occurs at multiple levels. One is the ‘between system’ decision to select one of the action maps out of the animal's behavioural repertoire, and as noted earlier this may be a major role of the basal ganglia [66,149]. The second is the ‘within system’ decisions that fine-tune the parameters of the action, which unfold within the associated cortical map [66,165]. For example, specification of different objects that afford reaching appears to take place within a population of tuned cortical neurons in the fronto-parietal reaching system (MIP, PMd, M1) [193,194,198,199]. These implement a ‘desirability density function’ across the space of potential reaching actions [207,208] and when multiple actions are present, a biased competition between them plays out across that population, possibly through lateral interactions between groups of cells that are selective for different reach directions [180,209,210]. Several lines of evidence suggest that when choosing specific movements within a given class of actions, it is the cortex that makes the choice [180,211,212] and not the basal ganglia [213–215].

In addition to the expansion of fronto-parietal action maps in primates, there was also a significant expansion of the temporal region. As in ancestral mammals, this region receives input from the primary visual cortex as well as collothalamic input from the lateral posterior thalamus (the pulvinar in primates) and uses it to detect particular combinations of features that, further downstream, can be combined to identify behaviourally relevant key stimuli. In primates, however, it goes far beyond simple key stimulus detection, classifying objects in terms of categories that reliably motivate a specific constellation of action opportunities.

Finally, of course, primates also dramatically expanded their frontal cortex. Passingham & Wise [16] suggest that this involved two major steps. The first took place about 70 Ma in the common ancestor of primates, which introduced granular orbitofrontal and caudo-lateral prefrontal areas. The second took place more recently, about 45 Ma in anthropoid monkeys, further parcellating frontal regions into dorsomedial, dorsolateral, ventrolateral and ventromedial prefrontal cortex as well as the frontal pole. Passingham & Wise [16] (see also [17]) propose that these steps made possible the more sophisticated foraging strategies that enabled primates to make the best use of their arboreal niche and in some lineages to return to terrestrial foraging.

In particular, living in trees is great for avoiding large predators, but it requires smarter choices about where and when to seek food. Travelling from branch to branch or tree to tree incurs risk, so it should be done only when one is reasonably sure the pay-off is worthwhile. Thus, searching for a food source is best done at a distance, orienting those large frontally facing eyes to focus on specific items using another innovation, which appeared in haplorhine monkeys—the fovea. This was a key function of the gaze control circuit, including area 8 and the FEF in the caudo-lateral prefrontal cortex [205,216]. Once a specific item is fixated, its desirability can be evaluated using the sophisticated object classification mechanisms in the temporal lobe and their projections into granular orbitofrontal cortex and ventral parts of prefrontal cortex [217,218]. If the fixated item is deemed desirable, i.e. its ‘offer value’ is high enough to warrant investment of time and energy, then one can engage the locomotion circuits, followed by the circuits for reaching, grasping and bringing the item to the mouth. If not, then one can simply move the eyes to consider another option. In summary, whereas early mammals foraged by physically running around and relying on olfaction to sniff out potential food sources, anthropoid primates accomplished similar tasks by relying more on vision—looking around and appraising fixated items and estimating the value of fruit-bearing trees from a distance, thus reducing exposure to threats.

The primate strategy of orienting the eyes towards objects of interest, prior to acting upon them, conferred an important ‘executive’ role to the gaze orientation system. Because most of the other fronto-parietal action systems relied so much on the high-resolution visual information from the fovea, selecting where to gaze now became part of selecting what to reach, or which branch to walk upon. In other words, it now took on a role often ascribed to the psychological construct of ‘attention’ [68]. Indeed it has long been proposed that selective attention, both overt and covert, is closely related to the gaze orientation system and involves the same neural structures [219,220], including the posterior parietal cortex, the frontal eye fields, and the superior colliculus. Hayden [179] proposes that the kinds of simultaneous choice tasks often used to study economic decision-making in the laboratory are actually solved by primates through a sequential strategy of fixating an object and then making a decision about whether to accept that offer or to reject it and look at other choices. In this way, the locomotor foraging strategies of early mammals may have become extended into attentional foraging strategies and more deliberative choices.

Finally, the further expansion of prefrontal cortex extended ancestral sensorimotor control towards higher levels of abstraction. Pezzulo & Cisek [208] propose that in addition to the affordances that are immediately present in the world, primates were able to predict what affordances would be made available as a consequence of their actions. For example, if a desirable fruit is out of reach, then walking forwards or pulling on a compliant branch will put it within reach. Consequently, one can weigh the available actions (e.g. walking forwards, pulling on a branch) by the predicted value of the currently unavailable reaching and ingesting action that they make possible. As noted earlier, the cerebellum has been strongly implicated in the prediction of action consequences, and in our recent evolutionary history, its expansion has been particularly prominent in the regions that are interconnected with prefrontal cortex [221]. Thus, the circuits originally adapted for immediate and real-time sensorimotor control could have gradually become extended towards increasingly temporally distant control goals.

## 7. Concluding remarks

The hypothetical sequence of behavioural and neuroanatomical elaborations described above expresses the general proposal that behaviour can be understood as feedback control [2–6] and that the history of brain evolution is the extension of that control further and further into the world through a process of continuous refinement [8]. According to this view, the neural organization of ancestral systems was gradually expanded along a given lineage into increasingly differentiated and elaborated systems, whose neuroanatomical substrates and functional roles remain at least partially distinguishable in descendant species. This paper outlines a particular hypothesis on how that process unfolded along the lineage from early eumetazoans to primates. Although my proposed sequence is likely to be mistaken in some important details, I would argue that some kind of sequence like this will ultimately be necessary to understand the human brain [8].

I have proposed that the functional architecture of primate brains can be thought of as a hierarchy of parallel competing control systems [13,176] whose organization is largely the product of the particular path our ancestors have taken through evolutionary history [8]. Atop the hierarchy is the hypothalamic control of basic physiological state, and below that a variety of systems for navigating around and interacting with the world to help keep the organism in a desirable state. Because of the complexities of interactive behaviour, these regions have expanded dramatically, rising and falling in prominence during particular niche transitions (e.g. reduction of tectal control in nocturnal mammals; expansion of eulaminate neocortex in arboreal primates). I claim that it is much easier to make sense of the organization of primate brains if we have at least a rough sketch of their evolutionary history, in part because it helps to identify the real underlying functional distinctions [8]. In other words, we should not assume that the brain decomposes into the sub-functions we have inherited from philosophical ideas about the human mind, and then search for their neural implementation in a brain whose architecture evolved millions of years before humans existed. Instead, we can decompose it into specific neural systems and the behavioural capacities they made possible at particular empirically definable transitions in our evolutionary history. Such a decomposition, I would argue, maps more readily to neural mechanisms than the classical frameworks of cognitive psychology [8,13,176], whose constructs are notoriously difficult to relate to neural data [13,18,222].

Of course, the theoretical framework outlined here is far from being able to explain the brain that interests us most, our own. At best, what I have described is just a rough sketch of a plausible candidate architecture of an anthropoid non-human primate. However, like any species, humans traversed their own path in evolution, and its twists and turns led to specific innovations in behavioural capacities and the brain circuits that support them. For example, modern human social systems and communication abilities are clearly far more complex than those of any other animal and involve specialized strategies and concomitant neural circuit properties. Understanding those from an evolutionary perspective is challenging because some of the most impressive adaptations almost certainly occurred long after we diverged from our nearest living cousins. Therefore, one may ask: does an evolutionary perspective offer anything towards understanding human-specific adaptations?

I believe that it does. Despite the dramatic differences in behavioural abilities and lifestyles, the human brain is remarkably similar to that of other primates in terms of the major topological organization, and it is likely that the most valuable insights into its peculiarities will come from studies on exactly where and how it differs [223–226]. In other words, whatever it was that happened in the last seven million years since our divergence from chimpanzees, it happened as further elaborations and specializations of the neural architecture of our last common ancestor. Understanding those adaptations can only be done in the context of the ancestral architecture they modified. Thus, although I admit that the framework outlined here is merely a candidate theory for explaining simple primate behaviour, I would propose that it provides a baseline set of constraints for any theory of human cognition.

## Data accessibility

This article has no additional data.
